# Outcomes of occipitocervical fixation using a spinous process screw in C2 as a third anchor point for occipitocervical fixation: a case presentation

**DOI:** 10.1186/s12891-020-03258-6

**Published:** 2020-05-16

**Authors:** Guanyi Liu, Qing Li, Feng Sheng, Nanjian Xu, Ming Li, Yang Wang, Weihu Ma

**Affiliations:** 1 Department of Orthopedics, Ningbo NO.6 Hospital, 1059 Zhongsandong Road, Ningbo, Zhejiang, 315040 People’s Republic of China; 2 Department of Endocrinology, Ningbo Yinzhou NO.2 Hospital, 998 Qianhebei Road, Ningbo, Zhejiang, 315000 People’s Republic of China

**Keywords:** Case report, International fixation, Cervical spine, Spinous process screw

## Abstract

**Background:**

Posterior occipitocervical fixation and fusion are often required to address occipitocervical instability. Safe, stable internal fixation with screws is vital for the success of such surgery. Thus, poor selection of an internal fixation technique may cause fixation and fusion failure, possibly leading to neurovascular injury. Hence, in certain cases, such as in patients with severe instability of an occipitocervical deformity or osteoporosis, we hypothesized that having a third anchor point (a screw in C2) could enhance the stability of the occipitocervical fixation.

**Case presentation:**

A 31-year-old man with occipitocervical deformity and spinal cord edema underwent a traditional occipitocervical fixation procedure but with the addition of a spinous process screw in C2 as a third anchor point. The procedure included posterior internal fixation and fusion. The occipitocervical fixation was completed by inserting occipital screws, bilateral C2 pedicle screws, C3 lateral mass screws, and a spinous process screw in C2 as a third anchor point. There were no neurovascular complications or incision-site infections. Postoperatively, radiography and computed tomography showed that the occipitocervical reduction and internal fixation had resulted in good spinal alignment, and magnetic resonance imaging showed no obvious spinal cord compression. At 4 months after the surgery, fusion was observed, and the occipitocervical screws remained well positioned. The patient continued to be monitored for 24 months postoperatively. At the 24-month follow-up visit, the muscle strength of the limbs was grade 5, and the patient’s sensation function had improved over his preoperative condition.

**Conclusions:**

Use of a C2 spinous process screw as a third anchor point may enhance the stability of occipitocervical fixation. Further biomechanical and clinical studies are needed to validate this result.

## Background

Degeneration, deformities, trauma, tumors, and other diseases can lead to occipitocervical instability, which often requires posterior occipitocervical fixation and fusion [1, 2]. When using the posterior approach to the occipitocervical joint, however, the anatomical structure and the presence of adjacent structures are complex and often result in shear stress concentration. Safe, stable internal fixation of the screws is vital for successful surgery. Poor selection of an internal fixation technique may cause the fixation and fusion to fail, possibly leading to neurovascular injury. The C2 vertebra is a commonly used anchor point for screws for both posterior upper cervical vertebral and occipitocervical fixation. The use of pedicle screws in C2 for fixation is relatively well established and commonly used, whereas the use of isthmus or lamina screws is less common [3, 4].

**Fig. 1:**
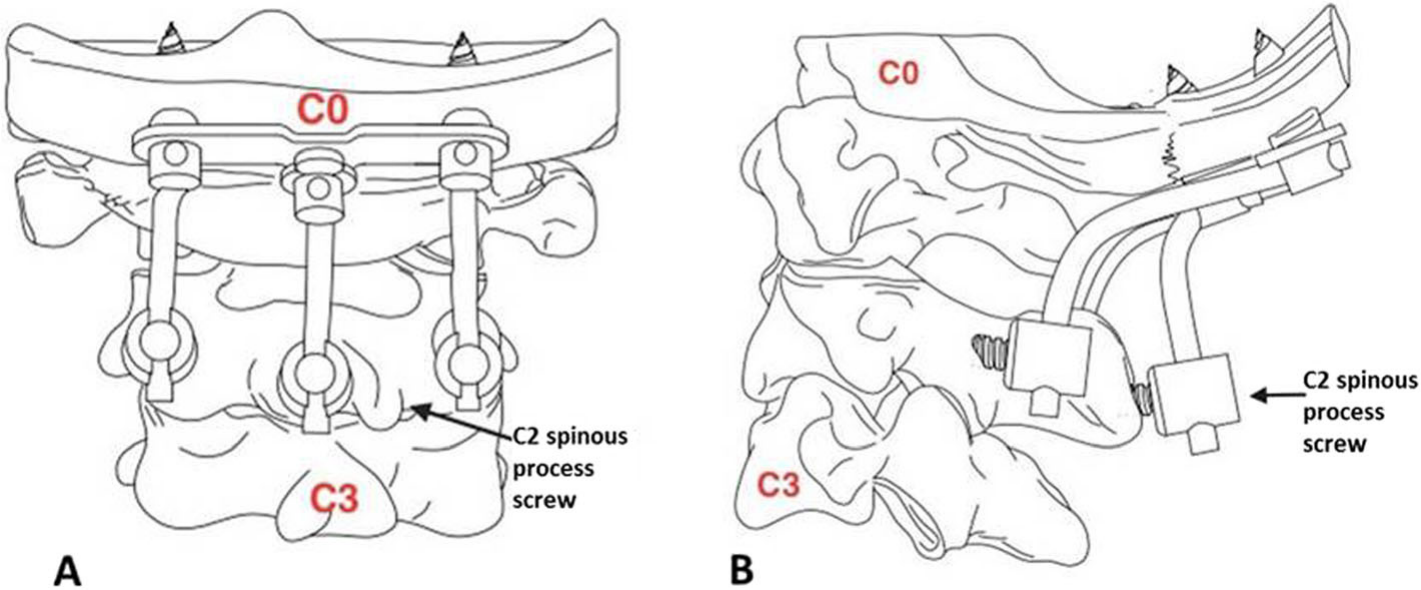
Spinous process screw as a third anchor point in C2 for occipitocervical fixation. **a** Anteroposterior view. **b** Lateral view

We therefore hypothesized that, in certain cases (e.g., patients with osteoporosis or severe instability of an occipitocervical deformity), placing a screw in C2 to establish a third anchor point could enhance the stability of the occipitocervical fixation. Hence, with the approval of the ethics committee of our hospital, we inserted an additional spinous process screw in C2 as a third anchor point for occipitocervical fixation in combination with bilateral pedicle screws (Fig. 1). This approach proved successful.

## Case presentation

**Fig. 2:**
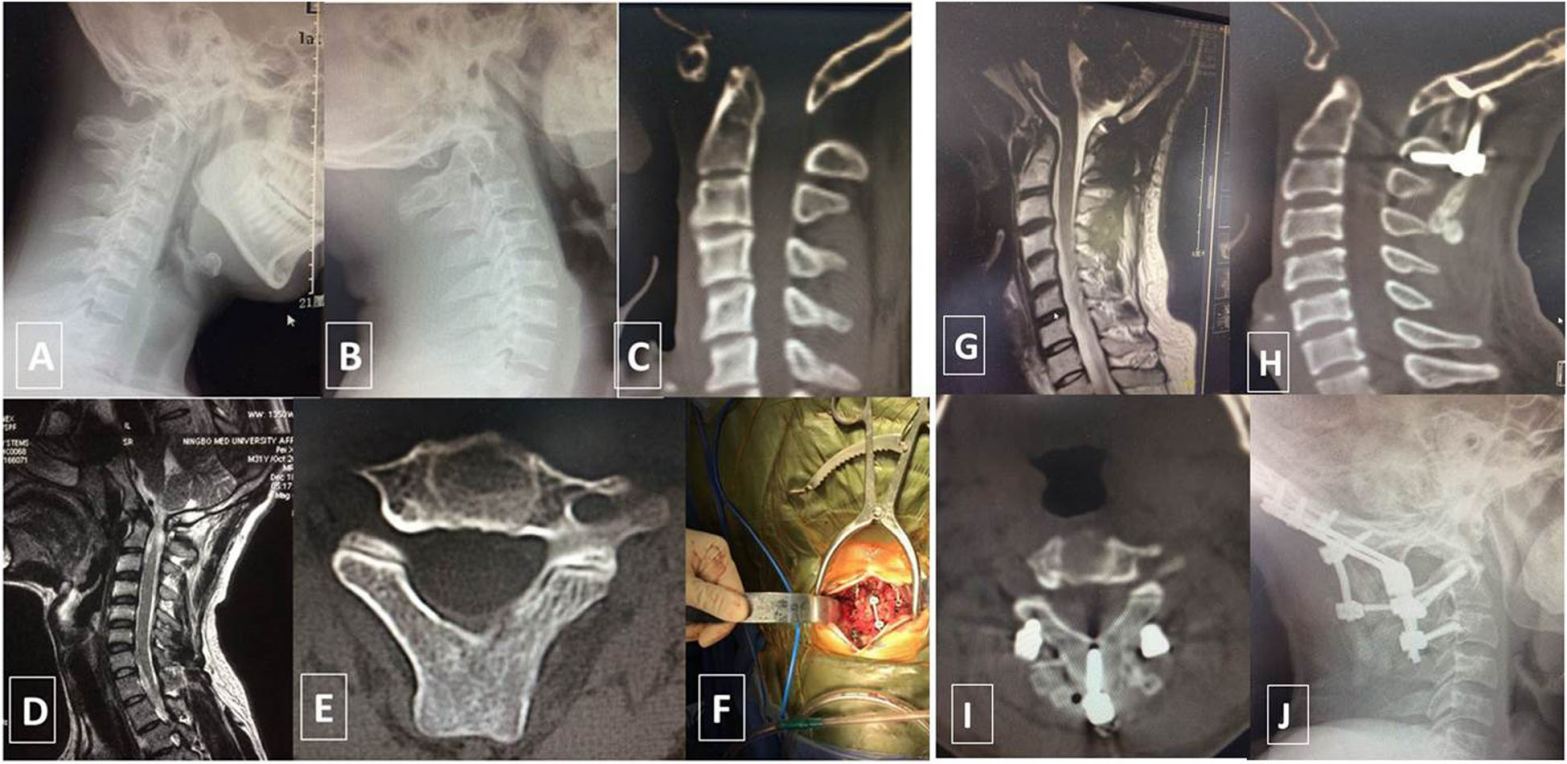
A 35-year-old man with occipitocervical deformity was treated with posterior occipitocervical fixation and fusion. **a–c** Occipital screws, C2 bilateral pedicle screws, a C2 spinous process screw, and C3 bilateral lateral mass screws were placed. Preoperative dynamic radiography and computed tomography (CT) reconstruction images show the occipitocervical deformity and instability. **d** Preoperative magnetic resonance imaging (MRI) shows the occipitocervical deformity and spinal cord compression. **e** Preoperative CT shows that the C2 spinous process was relatively broad and thick. **f** Intraoperative image shows the good position of the screws. **g** Postoperative MRI shows full decompression of the occipitocervical region. **h**, **i** Postoperative CT shows that the C2 spinous process screw was placed correctly, achieving successful occipitocervical fixation. **j** Follow-up lateral-view radiography shows that a good occipitocervical sequence was achieved, with no loosening of the internal fixation

A 35-year-old man was hospitalized after a 10-year history of occipitocervical pain and limited mobility (Fig. 2). Physical examination revealed mild tenderness in the back of the upper cervical vertebrae and limited mobility of the neck. The muscle strength of the limbs was grade 4, and there was slight loss of limb and trunk sensation. Excretory function was normal. Pathological reflexes were positive, and bilateral patellar tendon reflexes were hyperactive. Cervical anteroposterior and lateral plain radiography, cervical computed tomography (CT), and magnetic resonance imaging (MRI) revealed an occipitocervical junction deformity accompanied by spinal cord compression. Occipitocervical deformity was diagnosed. On the fifth day after admission, the patient underwent a corrective operation involving posterior reduction and occipitocervical fixation and fusion. The bone graft comprised both ilium and artificial bone.

The standard occipitocervical posterior midline approach was adopted to expose the posterior structures of the C0–C3 vertebrae. Care was taken to protect the vertebral artery and posterior vascular plexus. Intraoperative fluoroscopy revealed that the occipitocervical joint sequence was good, and the reduction was successful. Screw insertion was a three-step process. First, occipital screws were inserted in the mediolateral tubercle and the occipital protrusion of the occipital bone (total of three occipital screws) [5]. Second, pedicle screws were inserted bilaterally in C2 using the Harms and Melcher technique [6], and lateral mass screws were inserted bilaterally in C3 using the Roy–Camille et al. technique [7]. Third, an additional spinous process screw (3.5 mm diameter, 20 mm long) was inserted vertically into C2 [8]. The depth and angle of its implantation was determined using the Goel and Kulkarni technique [8], and the screw itself was chosen according to its length and diameter based on the patient’s appearance on preoperative CT scans. Following screw insertion, three rods were then connected from C0 to C3. An occipital screw was placed in the occipital protrusion and connected to the C2 spinous process screw. A high-speed drill was used to prepare the bone graft bed on the posterior structure of the occipitocervical joint. The graft comprised bone from the posterosuperior ilium and artificial bone.

**Fig. 3:**
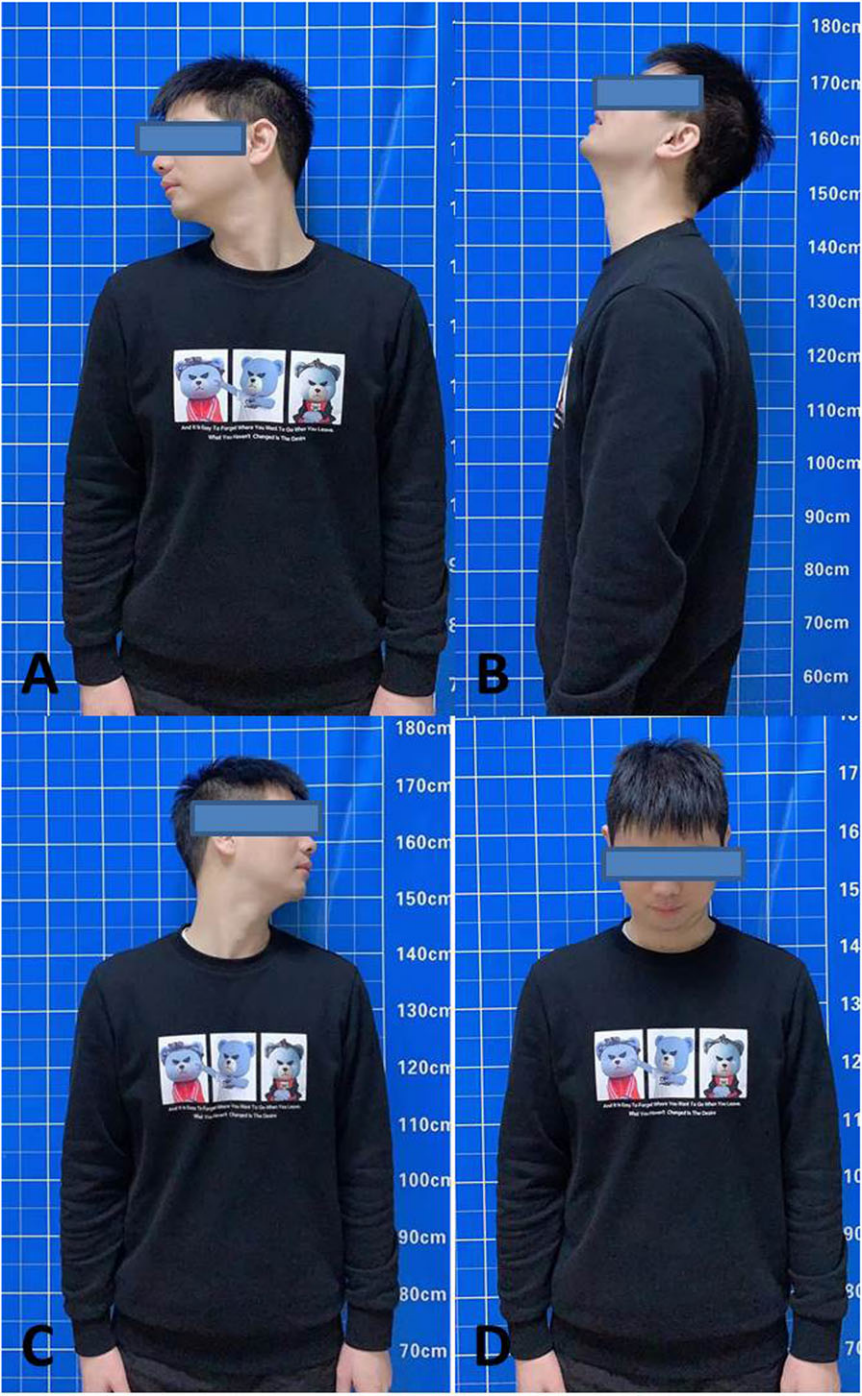
**a–d** Clinical photographs show the good cervical function of the patient in various positions at the 24-month follow-up visit

The patient could walk with a neck brace on postoperative day 3 and continued wearing the neck brace for the next 3 months. No neurovascular complications or incision infection were observed during the 24-month follow-up. Postoperative radiography showed that the internal fixation remained stable. Successful fusion was confirmed 4 months after the operation. Postoperative MRI showed that the occipitocervical decompression was sufficient, and there was no obvious spinal cord compression. The muscle strength of the limbs was grade 5, and at the 24-month follow-up visit the patient’s sensation function had improved from its preoperative state (Fig. 3).

## Discussion and conclusions

Use of a spinous process screw in C2 appears to be uncommon, with only a few clinical cases having been reported [8, 9]. This approach is mainly used as a supplementary method when there is an anatomical variation of the vertebral artery or pedicle or when other factors interfere with placement of a pedicle screw or some other, conventionally used screw [8–11]. One anatomical study showed that the average height of the C2 spinous process is about 12.9 mm, and the average thickness is 18.8 mm, indicating that screw fixation at this location is feasible [10]. In a biomechanical study, we found that the average pullout strength of a spinous process screw from C2 was 387 N, which is slightly lower than that of a pedicle screw (465 N) but without a statistically significant difference [11]. Dou et al. [12], based on results of an anatomical and mechanical study, came to a similar conclusion.

These findings led us to conclude that insertion of a spinous process screw in C2 may be useful as a third anchor point to achieve multi-screw occipitocervical fixation in cases of severe occipitocervical instability (even more so in osteoporotic and older patients). In 2004, Goel and Kulkarni [8] successfully performed vertical fixation with spinous process screws in C2 vertebrae in 11 children with congenital basilar invagination. Only one of the spinous process screws receded, although a second operation was not needed. Therefore, the authors concluded that a spinous process screw in C2 may be a reliable option for occipitocervical fixation.

The addition of a third anchor point, created by inserting a spinous process screw in C2, may stabilize the structures and shorten the cervical segments during occipitocervical fixation. It is controversial whether the C3 or C4 segment should be included in the occipitocervical fixation [13]. Martin et al. [14] reported that C0–C2–C3 occipitocervical fixation does not result in better stability than C0–C2 fixation, and the addition of C3 lateral mass screws may increase the risk of vertebral artery injury and cervical segment loss. In the present case, a spinous process screw was inserted in C2 to be used as a third anchor point in combination with bilateral pedicle screws for C2 fixation. This combination may increase fixation strength and, thereby, reduce the need to extend the fixation to C4.

Although the use of a C2 spinous process screw may be effective for reinforcing occipitocervical fixation, it has inherent drawbacks. For example, inserting a spinous process screw is a simple approach to posterior column fixation, but it is not suitable for hangman’s fractures. It may also affect the bone graft area used in posterior fusion procedures and introduce the risk of violating the spinal canal. Surgical contraindications mainly include fractures of the lamina and spinous process—conditions necessitating removal of the spinous process—and other conditions that would make the environment unsuitable for fixation. Thus, further biomechanical studies on the implantation of a C2 spinous process screw and how it affects the biomechanics and stability of pedicle screws used in occipitocervical fixation are needed before this approach can be advocated for wide use.

Thus, a C2 spinous process screw inserted as a third anchor point may enhance the stability of occipitocervical fixation. Nevertheless, further biomechanical and clinical studies are needed.

## Data Availability

The data sets used and/or analyzed during the current study are available from the corresponding author on reasonable request.

## Abbreviations

CT: Computed tomography
MRI: Magnetic resonance imaging
